# Discovery of Specific Metastasis-Related *N*-Glycan Alterations in Epithelial Ovarian Cancer Based on Quantitative Glycomics

**DOI:** 10.1371/journal.pone.0087978

**Published:** 2014-02-06

**Authors:** Xingwang Zhang, Yisheng Wang, Yifan Qian, Xin Wu, Zejian Zhang, Xijun Liu, Ran Zhao, Lei Zhou, Yuanyuan Ruan, Jiejie Xu, Haiou Liu, Shifang Ren, Congjian Xu, Jianxin Gu

**Affiliations:** 1 Key Laboratory of Glycoconjugate Research Ministry of Public Health, Department of Biochemistry and Molecular Biology, School of Basic Medical Sciences, Fudan University, Shanghai, China; 2 Obstetrics and Gynecology Hospital, Fudan University, Shanghai, China; 3 Department of Obstetrics and Gynecology of Shanghai Medical School, Fudan University, Shanghai, China; 4 Institute of Biomedical Sciences, Fudan University, Shanghai, China; 5 Shanghai Key Laboratory of Female Reproductive Endocrine Related Diseases, Shanghai, China; University of Alabama at Birmingham, United States of America

## Abstract

Generally, most of ovarian cancer cannot be detected until large scale and remote metastasis occurs, which is the major cause of high mortality in ovarian cancer. Therefore, it is urgent to discover metastasis-related biomarkers for the detection of ovarian cancer in its occult metastasis stage. Altered glycosylation is a universal feature of malignancy and certain types of glycan structures are well-known markers for tumor progressions. Thus, this study aimed to reveal specific changes of *N*-glycans in the secretome of the metastatic ovarian cancer. We employed a quantitative glycomics approach based on metabolic stable isotope labeling to compare the differential *N*-glycosylation of secretome between an ovarian cancer cell line SKOV3 and its high metastatic derivative SKOV3-ip. Intriguingly, among total 17 *N*-glycans identified, the *N*-glycans with bisecting GlcNAc were all significantly decreased in SKOV3-ip in comparison to SKOV3. This alteration in bisecting GlcNAc glycoforms as well as its corresponding association with ovarian cancer metastatic behavior was further validated at the glycotransferase level with multiple techniques including real-time PCR, western blotting, transwell assay, lectin blotting and immunohistochemistry analysis. This study illustrated metastasis-related *N*-glycan alterations in ovarian cancer secretome *in vitro* for the first time, which is a valuable source for biomarker discovery as well. Moreover, *N*-glycans with bisecting GlcNAc shed light on the detection of ovarian cancer in early peritoneal metastasis stage which may accordingly improve the prognosis of ovarian cancer patients.

## Introduction

Epithelial ovarian cancer is the most lethal gynecological malignancy worldwide [1], [2]. According to a recent report of the American Cancer Society, approximately 22,240 new cases will be diagnosed with ovarian cancer in 2013 while nearly 14,030 will succumb to this disease [3]. Currently, pelvic ultrasonography and serum CA125 test serve as main detection routines [4], [5], [6], [7], which are effective in some cases. However, the majority of ovarian cancer may still not be diagnosed until the tumor has progressed to an advanced stage, presenting with large-scale or remote metastasis. In the clinic, the most common metastatic pattern of ovarian cancer is recognized as peritoneal implantation [8]. Due to the fact that early peritoneal metastasis sites are usually too small to be detected macroscopically [4], [9], the five-year survival rate always suffers this poor terminal diagnosis and witnesses a dramatical decrease when large-scale peritoneal metastasis occurs, from about 90% at stage I to 2–80% at stage II-IV [4], [10], [11]. Therefore, it is necessary to discover metastasis-related biomarker which could aid in the detection of ovarian cancer as early as possible rather than until the wide spread of tumor outside the ovaries.

Glycosylation is one of the most prevalent post-translational modifications which produces various types of glycans that are frequently attached to proteins. Glycans participate in major pathophysiology events during tumor progressions [12], [13], [14]. Altered glycosylation is a universal feature of malignancy, and some of these alterations contribute to metastatic processes [12], [15]. For instance, increased activity or expression of *N*-acetylglucosaminyltransferase V (MGAT5) and β-1, 6 GlcNAc-branched *N*-glycans has been found in highly metastatic tumors, such as colon, hepatic and breast cancers [16], [17], [18], [19], [20]. Additionally, the alteration in sialylation has been reported contributing to the metastasis in these tumor cells as well [21], [22], [23], [24], [25], [26], [27]. Moreover, other aberrant glycosylation patterns including core-fucose, bisecting GlcNAc *N*-glycans have been known to be associated with metastasis of many malignant cells [28], [29], [30], [31], [32]. Accordingly, cancer-associated aberrations in glycan structures provide a compelling rationale for new biomarkers discovery [33].

In the case of ovarian cancer, aberrant glycosylation has been described in multiple studies, mainly including increased silylaiton and fucosylation of *O*-linked glycans, higher levels of biantennary *N*-linked glycans, elevated core fucosylation, and bisecting *N*-linked glycosylation [34]. Currently, the role of glycosylation modification playing in ovarian cancer metastasis has also been indicated in several studies. For example, mesothelin-MUC16 binding that facilitates peritoneal metastasis of ovarian cancers has been shown *N*-glycan dependent [35]. The cell ability of adhesion to hyaluronan in ovarian carcinoma cell lines could be altered by removing carbohydrate moieties from the cell surfaces [36]. These studies suggested the involvement of glycans in the metastasis processes, whereas exact *N*-glycan changes associated with metastasis in ovarian cancer remained unclear, such as those on cell surface and cell secreted glycoproteins. Given that specific glycan structure changes in cancer cell secretome may be potential biomarkers for non-invasive diagnosis [37], in this study, we focused on revealing the metastasis-associated *N*-glycosylation alteration in glycoproteins secreted by ovarian cancer cells.

**Figure 1:**
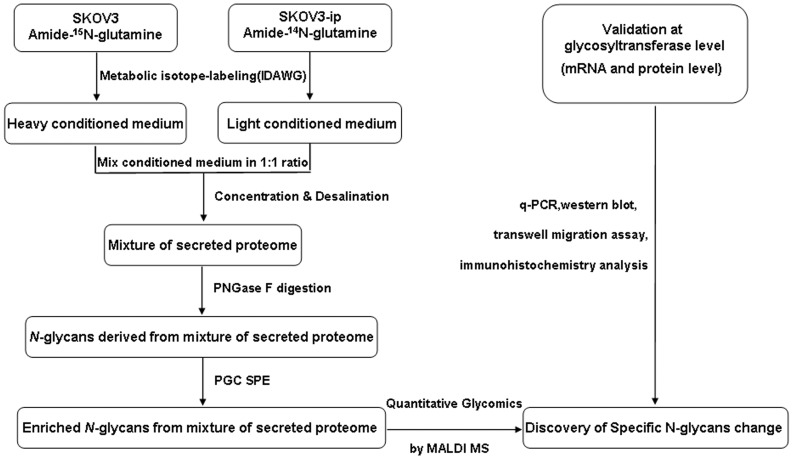
The total experimental workflow in this study.

Quantitative glycomics based on mass spectrometry (MS) has been proved to be a promising and powerful tool to identify global alterations of glycans between different biological samples [38], [39], [40], which includes label free strategies and stable isotope labeling strategies. The former one is much simpler while the latter one is more robust. One quantitative glycomics method based on metabolic incorporation of stable isotope labels (Isotopic Detection of Aminosugars With Glutamine, IDAWG) has been reported recently [41]. In this method, differentially labeled cells can be mixed together early in the work flow, which minimize any potential bias introduced during sample processing and offer a reliable quantification method [39], [42]. In our study, different from quantitative glycomics analysis of cell surface glycoproteins using IDAWG reported previously, quantitative glycomics method based on metabolic incorporation of stable isotope labels was employed to differential *N*-glycosylation analysis of secretome between two ovarian cancer cell lines, SKOV3 and its high metastatic derivative SKOV3-ip. Subsequently, metastasis-associated *N*-glycan changes in ovarian cancer discovered by this glycomics method were further validated at glycotransferase level with different molecular biological techniques, including real-time PCR, western blotting, lectin blotting, transwell assay and immunohistochemistry analysis. The total work flow in our study was shown in Figure 1. To our knowledge, this is the first attempt to reveal global picture of the metastasis-related *N*-glycan alterations in ovarian cancer cell secretome. Moreover, among these changes, decrease in bisecting GlcNAc modification and its corresponding glycotransferase (Mannosyl (Beta-1,4-)-Glycoprotein Beta-1,4-N-Acetylglucosaminyltransferase, MGAT3) expression were demonstrated to be related to higher metastatic potential. This study provided insights into discovery of metastasis-associated biomarker, which could aid in detecting early peritoneal metastasis of ovarian cancer, and finally improving diagnosis of ovarian cancer.

## Materials and Methods

### Cell Culture and Metabolic Stable Isotope Labeling

Serous ovarian cancer cell line SKOV3 and its high metastatic derivative SKOV3-ip were obtained from the American Type Culture Collection (ATCC). These two ovarian cancer cell lines were grown in RPMI1640 cell media (Invitrogen, Carlsbad, CA, USA) supplemented with 10% fetal bovine serum (Invitrogen). 293T cell line was obtained from the Institute of Cell Biology Academic Sinica (Shanghai, China). 293T cells were maintained in Dulbecco’s modified Eagle’s medium (DMEM) containing 10% fetal bovine serum. All cells were cultured with 1% penicillin/streptomycin at 37°C in a humidified 5% CO_2_ incubator. For stable isotope labeling experiments, cells were cultured for at least six doublings in the RPMI1640 medium with 10% fetal bovine serum, containing 20 mM L-glutamine (either ^14^N or amide-^15^N-Gln) to achieve complete incorporation of labeled *N*-glycans [41]. Amide-^15^N-Gln (98% purity) was purchased from Cambridge Isotopes, Inc. (Andover, MA, USA).

### Supernatant Acquisition

Six million labeled cells were seeded in 55-cm^2^ cell culture dishes and maintained overnight for cell attachment in the RPMI1640 medium supplemented with 10% fetal bovine serum, 20 mM L-glutamine (either ^14^N or amide-^15^N-Gln). After 12 h, the culture medium was removed, and the cells were washed for 3 times with phosphate-buffered saline. And then 10 mL of serum-free, phenol red-free RPMI1640 medium with 20 mM L-glutamine (either ^14^N or amide-^15^N) was added to each dish. After incubation for 48 h, the conditioned medium was collected and filtered through a 0.22 µm Durapore PVDF Membrane (Millipore, Billerica, MA, USA). Before, during and after serum starvation, viability of cancer cells was assessed using the trypan blue dye exclusion assay. The protein concentrations of conditioned media were determined using a BCA protein assay kit (Pierce,Rockford, IL). The conditioned medium with equal protein amount obtained from SKOV3 and SKOV3-ip were mixed. Subsequently, the mixtures were concentrated by Amicon Ultra-15, 3,000 molecular weight cut off (MWCO) centrifugal filter units (Millipore, Billerica, MA, USA) with excessive salt removing for quantitative glycomics. All samples were stored at −80°C until further use.

### Release and Purification of N-Glycans from Secreted Proteome

The *N*-linked glycan release and purification were performed as previously described with minor modifications [43]. Aliquots of mixed protein solution (100 µL) were diluted with equal volume of denaturing buffer composed of 200 mM NH_4_HCO_3_ (Sigma-Aldrich, Germany) containing 20 mM mdithiothreitol (Sigma-Aldrich, Germany). The samples were heated for 10 min in 95°C water bath. After the denatured samples cooled to the ambient room temperature, 1 µL of PNGase F (New England Biolabs, Inc., USA) was added to each sample followed by overnight incubation at 37°C. Subsequently, the deglycosylated proteins were precipitated by adding 600 µL of chilled ethanol stored at –80°C for 2 h. After centrifugation at 14,000×g, for 30 min at 4°C, the supernatant was collected and dried through vacuum centrifugation. The released glycans were further purified and desalted using a Porous Graphic Carbon Solid-Phase Extraction (PGC-SPE). To this end, the PGC microcolumns were packed with porous graphic carbon powder and prepared using GELoader tips as described before [44]. The microcolumns were preconditioned with 6 column volumes of 0.1% (v/v) trifluoroacetic acid (TFA) in 80% acetonitrile (ACN)/H_2_O (v/v) and equilibrated with equal volume of water. Afterwards, the samples were applied to the columns repeatedly for 5 times to achieve complete glycans adsorption. Subsequently, the microcolumns were washed with approximately 10 column volumes of water to remove salts and buffer. Glycans were eluted with 25% (v/v) ACN containing 0.05% (v/v) TFA. Each sample was then divided into two equal aliquots and lyophilized in a vacuum freeze dryer (Martin Christ GmbH, Osterode,Germany)**.** One equal aliquot of each sample was stored at –80°C until further analysis. Another equal aliquot of each sample was permethylated.

### Permethylation of *N*-glycans

*N*-glycans were permethylated using a previously published procedure with minor modifications [45]. Briefly, dried *N*-glycans were suspended in 200 µL of dimethyl sulfoxide (DMSO), and approximately 20 mg NaOH powder was added. After the slurry was mixed vigorously, 100 µL of CH_3_I was added and the following incubation was conducted in an ultrasonic bath for 30 min. The reaction was quenched by addition of 1 mL of water, and the excess CH_3_I was removed under a stream of nitrogen. Subsequently, 1 mL of chloroform was added with strong mixing. After phase separation, the aqueous phase was discarded while the lower chloroform phase was washed 10 times with 1 mL of H_2_O and dried down under a stream of nitrogen in a hood for matrix-assisted laser desorption/ionization mass spectrometry (MALDI MS) analysis.

### MALDI MS Analysis

MALDI mass spectrometric analysis was performed on AXIMA Resonance MALDI QIT TOF MS (Shimadzu Corp., Kyoto, Japan) with a 337 nm nitrogen laser. All samples were analyzed at a 20-kV accelerating voltage in the positive ion and reflectron mode. CID was performed at a collision energy of 4 keV using argon as the collision gas. The instrument was externally calibrated by TOFMix™ (LaserBio Labs, France) containing eight peptides calibration standard. Prior to MS analysis, unpermethylated glycans were reconstituted in a 5-µL aliquot of 0.1% (volume/volume, v/v) TFA in 50% ACN/H_2_O (v/v) and permethylated glycans were reconstituted in a 5-µL aliquot of 50% methanol. Each sample (1 µL) was spotted onto a MALDI target and allowed to dry in air at ambient temperature. Afterwards, 1 µL of 2,5-dihydroxybenzoic acid matrix (DHB, Sigma-Aldrich, Germany) at the concentration of 12.5 mg mL^-1^ in 50% ACN in water (v/v) containing 0.1% TFA was added onto the sample layer and dried under ambient conditions. Each sample was spotted in triplicate. Two laser shots were set to generate a profile and 200 profiles were accumulated from different points of laser irradiation into one file for each sample spot. For comparison of *N*-glycans from secretomes of the two ovarian cancer cells, a 1∶1 mixture of ^14^N- and ^15^N-labeled samples was initially obtained through mixing equal starting proteins amount. And the mixture was analyzed (three replicates) to determine relative changes in the abundances of *N*-glycans between ovarian cancer cell line SKOV3 and its high metastatic derivative SKOV3-ip. All the proposed structures of identified glycans were interpreted manually based on their *m/z* values according to serveral previously published literatures [46], [47], GlycoWorkbench [48], Glycan Mass Spectral Database [49] as well as GlycoBase (Version 2, http://glycobase.nibrt.ie/glycobase.html).

### Quantitative Real-time PCR for mRNA Expression Analysis

Total RNAs were extracted from ovarian cancer cell lines with the TRIzol™ reagent (Invitrogen) and 2 µg of total RNA was reverse transcribed using RT Master Mix kit (Takara) according to the manufacturer’s instruction. Real-time PCR was performed on ABI 7500 Fast Real-time PCR system (Applied Biosystems). The PCR cycling conditions consisted of a single incubation step at 95°C for 30 s, followed by 40 cycles of 5 s at 95°C, and 34 s at 60°C. All PCR reactions were performed with SYBR-green Premix Real-time PCR kit (Takara) according to the manufacturer’s protocol. Primers sequences used in PCR analysis were as follows: human MGAT3 forward (5′-CAGGGCACAGGATTGAAGAGTT-3′) and human MGAT3 reverse (5′-AGCTTGGTTTCCCTTCATATTGAG-3′); human MGAT5 forward (5′- GACCTGCAGTTCCTTCTTCG-3′) and human MGAT5 reverse (5′- CCATGGCAGAAGTCCTGTTT-3′); human Glyceraldehyde-3-phosphate dehydrogenase (GADPH) forward (5′-CTCTCTGCTCCTCCTGTTCGAC-3′) and human GAPDH reverse (5′-TGAGCGATGTGGCTCGGCT-3′). Human GADPH mRNA served as an endogenous control for normalization. Real-time PCR analysis was carried out in triplicate.

### Plasmids and Gene Overexpression

SKOV3-ip and SKOV3 cells were transfected using X-tremeGENE HP DNATransfection Reagent (Roche Applied Science, Mannheim, Germany) with pcDNA3.1-MGAT3 or pcDNA3.1-MGAT5 plasmids, respectively (provided by a researcher in our laboratory), according to the manufacturer’s instructions. And pcDNA3.1 vector was used as the negative control. At 48 h after transfection, the overexpression of MGAT3 and MGAT5 were confirmed by western blot analysis.

### Small Interfering RNA and Knockdown of MGAT3

SKOV3 cells were transfected with MGAT3 specific siRNA Transfection Reagent Complex, (Santa Cruz Biotech, Inc., sc-44469), which was prepared according to the manufacturer’s protocol. Scrambled siRNA was used as the negative control. At 48 h after transfection, cell lysates were prepared for western blot analysis to determine gene knockdown efficacy.

### Western Blot and Lectin Blot Analysis

In order to obtain whole cell proteins, the cells were collected, washed with phosphate buffer solution (PBS) and then lysed in SDS lysis buffer [50]. Total protein concentrations were determined by BCA assay (Pierce,Rockford, IL). Equal amounts of total protein lysates from each cell line were separated by 10% sodium dodecyl sulfate polyacrylamide gel electrophoresis (SDS-PAGE) and then transferred to PVDF membranes (Roche Applied Science). i. Western blot analysis: After blocking with 5% skimmed milk in Tris-buffered saline containing 0.1% Tween 20 (TBST) for 2 h at room temperature, the membranes were incubated overnight at 4°C with mouse monoclonal antibody against MGAT3 (1/1000 diluted, sigma) or MGAT5 (1/1000 diluted, sigma) and then a secondary antibody at room temperature for 1 h. After washing, the membranes were detected by enhanced chemiluminescent (ECL) assay kit. ii. Lectin blot analysis: After blocking with 5% BSA in TBST, the membranes were incubated overnight at 4°C with biotinylated *Phaseolus vulgaris erythroagglutinin* (E-PHA) (1/5000 diluted,Vector Labs) or *Phaseolus vulgaris leucoagglutinin* (L-PHA) (1/5000 diluted,Vector Labs). Next, membranes were washed four times with TBST, followed by incubation with horseradish peroxidase streptavidin (Vector Labs) for 30 min at room temperature. Subsequently, the membranes were washed four times with TBST and detected by ECL assay kit.

### Transwell Migration Assay

To evaluate the migratory ability, cell migration was performed using 24-well format transwell chambers (8 µm pore filter, Corning, Canton, NY). Briefly, SKOV3 cells were transfected with MGAT3 specific siRNA or MGAT5 plasmid, and SKOV3-ip cells were transfected with MGAT3 plasmid for 36 h, respectively. After that, cells were trypsinized, collected, counted, and then suspended in serum-free RPMI1640 medium. 2×10^4^ cells in 100 µL of serum-free RPMI1640 medium were platted to each insert of the upper chamber. Meanwhile, 600 µL of RPMI1640 medium containing 10% FBS was added to each lower chamber. Subsequently, the cells were incubated for 12 h at 37°C. After removing the cells on the upper membrane surface, cells on the bottom surface of the membrane were fixed and stained with 0.1% crystal violet for 30 min at room temperature. Next, cells that had passed through the filter to the lower chamber were counted microscopically in 6 random regions per filter. The number of cells that traversed the membrane revealed the migratory ability of the tested cells.

### Immunohistochemistry Analysis

A total of 24 ovarian cancer tissues were fixed in 4% formalin and embedded in paraffin. Tissue sections were first subjected to hematoxylin and eosin (H&E) staining for histopathological diagnosis. Then, immunohistochemical staining using anti-MGAT3 antibody (Abcam, Cambridge, MA, USA) was performed as described previously [51]. The intensity of MGAT3 staining was scaled from 0 to 3, where 0 stands for negative staining, 1, 2, 3 stand for weak, moderate, and strong staining respectively. Ovarian cancer tissues were obtained from the Obstetrics and Gynecology Hospital, Fudan University, Shanghai, China. All protocols have been approved by the Institutional Review Board of the hospital. All patients have given their written informed consent. Clinical information for these patients were shown in Table S1.

### Data Processing and Statistical Analysis

MALDI MS and tandem mass spectrometry (MS/MS) data were acquired and processed in Launchpad software (Shimadzu Biotech, Kyoto, Japan). The parameters of peak processing: smoothing method: Gaussian; peak detection method: threshold-25% centroid; threshold offset: 0.500 mV. The *m/z* values and intensities were exported as ASCII files and peak intensities were scaled with the highest peak as 100%. The relative quantification of detected *N*-glycans was according to the protocols previously reported [42]. All statistical analyses were performed using GraphPad Prism (version 5). All the experiments were performed at least three times. The data were expressed as the means ± standard error of the mean (SEM). Student’s *t*-test was used to determine the significance of differences between two groups. Wilcoxon Signed Ranks Test was used to compare immunohistochemistry data from the two groups. A *p*-value of less than 0.05 was considered to be statistically significant.

## Results

### Quantitative/Comparative Glycomics Analysis of Supernatant between SKOV3 and SKOV3-ip Cell

**Figure 2:**
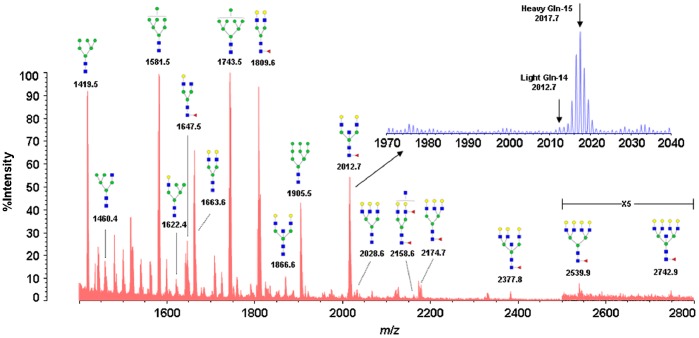
Quantitative analysis of *N*-linked glycans using MALDI-TOF MS. *N*-linked glycans from a 1∶1 mixture of SKOV3-ip (Gln-14 labeled) and SKOV3 (Gln-15 labeled) supernatant. The structures were interpreted manually based on their *m/z* values according to several previously published literatures [46], [47], GlycoWorkbench [48] as well as Glycan Mass Spectral Database [49]. Gln-14 indicates amide-^14^N-Gln; Gln-15 indicates amide-^15^N-Gln. Green circle indicates mannose; yellow circle indicates galactose; blue square indicates *N*-acetylglucosamine; red triangle indicates fucose.

To discover the metastasis-related *N*-glycan alteration in ovarian cancer cell secretome, the total pool of *N*-glycan mixture, released by the enzyme Peptide *N*-glycosidase F from the secretome mixture of SKOV3(amide-^15^N-Gln labeled) and SKOV3-ip cells (amide-^14^N-Gln labeled), was analyzed by MALDI-QIT-TOF MS (Figure 2). A total of 17 *N*-glycans were identified, including high mannose, hybrid, triantennary, tetra-antennary structures and bisecting GlcNAc glycoforms. All the identified *N*-glycans with proposed structure were shown in Figure 2 and summarized in Table 1.

**Table 1:** Relative quantitation of *N*-glycans detected in SKOV3-ip (amide-^14^N-Gln-labeled) and SKOV3 (amide-^15^N-Gln-labeled) supernatant.

No.	Observed *m/z* [M+Na]^+^	Theoretical *m/z* [M+Na]^+^	Ratio (SKOV3-ip/SKOV3)	CV (%) (n = 9)	Change^a^	Composition	Type
1	1419.5	1419.5	1.46	4.26	up	HexNAc_2_Hex_6_	High mannose
2	1460.4	1460.5	2.05	13.44	up	HexNAc_3_Hex_5_	Hybrid
3	1581.5	1581.5	0.95	3.49	no	HexNAc_2_Hex_7_	High mannose
4	1622.4	1622.6	1.63	12.61	up	HexNAc_3_Hex_6_	Hybrid
5	1647.5	1647.6	3.40	11.48	up	HexNAc_4_Hex_4_ Fuc	Complex biantennary
6	1663.6	1663.6	3.23	8.78	up	HexNAc_4_Hex_5_	Complex biantennary
7	1743.5	1743.6	1.42	4.29	up	HexNAc_2_Hex_8_	High mannose
8	1809.6	1809.6	2.21	8.87	up	HexNAc_4_Hex_5_ Fuc	Complex biantennary
9	1866.6	1866.7	0.28	2.40	down	HexNAc_5_Hex_5_	Bisecting
10	1905.5	1905.6	2.19	10.01	up	HexNAc_2_Hex_9_	High mannose
11	2012.7	2012.7	0.12	15.20	down	HexNAc_5_Hex_5_ Fuc	Bisecting
12	2028.6	2028.7	1.32	18.06	up	HexNAc_5_Hex_6_	Triantennary
13	2158.6	2158.8	0.64	17.46	down	HexNAc_5_Hex_5_ Fuc_2_	Complex biantennary
14	2174.7	2174.8	2.21	15.99	up	HexNAc_5_Hex_6_ Fuc	Triantennary
15	2377.8	2377.9	0.32	9.87	down	HexNAc_6_Hex_6_ Fuc	Bisecting
16	2539.9	2539.9	1.78	15.90	up	HexNAc_6_Hex_7_ Fuc	Tetra-antennary
17	2742.9	2743.0	0.73	15.35	down	HexNAc_7_Hex_7_ Fuc	Bisecting

a Ratio (SKOV3-ip/SKOV3) <0.83 or >1.20 is distinguishable as a criterion to estimate the down- or up-regulation of the glycan levels. HexNAc, N-acetylhexosamine; Hex, hexose (mannose or galactose ); Fuc, fucose.

Quantitative analysis of *N*-glycans from the secretome mixture of SKOV3 and SKOV3-ip cells was based on signal intensities. Intensity ratios (SKOV3-ip/SKOV3) of 0.83 and 1.2 were set as the threshold to estimate the down- or up-regulation of the *N*-glycan levels in SKOV3-ip cells, in comparison to SKOV3 cells, since the maximum coefficient of variation (CV) (n = 9) was below 20% in this study [43]. Relative quantitation of all detected *N*-glycans between SKOV3-ip and SKOV3 cells were summarized in Table 1. The results demonstrated that three out of four high mannose structures were up-regulated in SKOV3-ip cells. *N*-glycans containing hybrid, triantennary, and tetra-antennary structures with or without fucose were elevated in SKOV3-ip cells. The similar trend was observed in three glycans with complex biantennary structures. Among all the alteration in *N*-glycans, all the *N*-glycans with bisecting GlcNAc were found to be apparently decreased in the SKOV3-ip cells, with their SKOV3-ip/SKOV3 ratios ranging from 0.73 to 0.12, while all the β-1,6 GlcNAc-branched *N*-glycans were elevated in SKOV3-ip cells. Among these altered *N*-glycans, we focused on the *N*-glycans with bisecting GlcNAc, partly because all of them decreased and they were the most obviously altered *N*-glycans. Furthermore, an increasing body of evidence has indicated that the bisecting glycans may affect tumor progression and metastasis [52].The structures containing bisecting GlcNAc were further confirmed by tandem mass spectrometry (MS/MS). Representative tandem MS spectra of *m/z* 2012.7 [M+Na]^+^, and *m/z* 2377.8 [M+Na]^+^ were shown in Figures S1A–B.

It is noteworthy that sialic acids, typically presented as terminal monosaccharides in the glycan moiety of many glycoproteins, play essential roles in many physiological and pathological processes. Sialylated glycans are labile and easy to be lost during direct mass spectrometric analysis of underivatized *N*-glycans. As permethylation can stabilize the sialic acid residues by converting highly polar -OH and -COO^−^ groups of the different monosaccharides into nonpolar [38], *N*-glycans after permethylation were also analyzed. The results demonstrated that more *N*-glycans with different degree of sialylation were observed comparing to the analysis results without permethylation. Although signal of one *N*-glycan with bisecting GlcNAc could be decentralized to several signals of *N*-glycans with different degree of sialylation, the trend of alteration in all the bisecting GlcNAc structures were the same no matter permethylated or not (see Figure S2). The signal intensity of the bisecting *N*-glycan without permethylation (Figure S2A, *m/z* 2012.6 for example) was higher than that of permethylated *N*-glycans at *m/z* 2489.4, *m/z* 2850.3 and *m/z* 3211.4 containing 0, 1 and 2 sialic acids respectively (Figure S2B). It is possibly because part of the original bisecting signal intensity (Figure S2A) was contributed to the corresponding fragments of the glycans with same structure while containing additional 1 and 2 sialic acids (Figure S2B). Furthermore, the glycan profiles with permethylation were more complex (i.e.,have more peaks). Thereby, it is suggested that the permethylation step probably could be omitted in quantitative glycomics analysis of those neutral *N*-glycans which is irrelative to sialic acids, such as bisecting GlcNAc glycoform in this study.

### Differential Expression of MGAT3 in SKOV3-ip and SKOV3 Cell Lines

**Figure 3:**
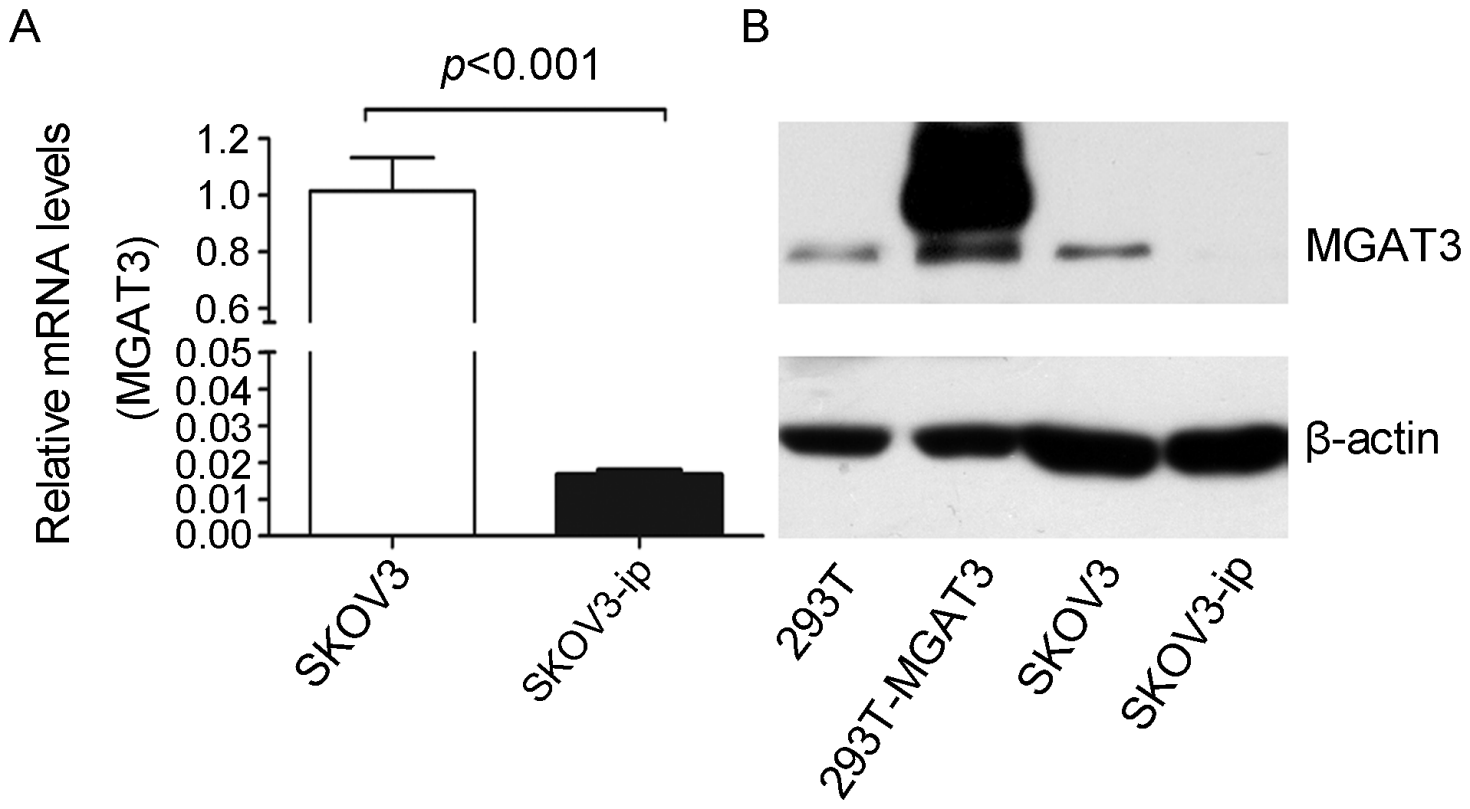
Differential expression of MGAT3 in SKOV3-ip and SKOV3 cell lines. (A) The mRNA expression levels of MGAT3 in SKOV3-ip and SKOV3 cell lines. The mRNA levels of MGAT3 were normalized with GAPDH levels. Mean fold changes were calculated with the 2^–ΔΔCt^ method. (B) The protein expression levels of MGAT3 in SKOV3-ip and SKOV3 cell lines. Cell lysates were isolated by 10% SDS-PAGE for western blotting using antibody against MGAT3. Human beta-actin served as an endogenous control. 293T cell line was used as positive control. Data are expressed as the means ± SEM. Each assay was performed at least three times. A *p*-value of less than 0.05 indicates statistical significance using Student’s *t*-test.

As bisecting GlcNAc residue was synthesized by specific glycosytransferase (MGAT3), the change in *N*-glycans with bisecting GlcNAc could be correlated with the corresponding alteration in MGAT3. Therefore, real-time PCR analysis was carried out to compare the mRNA expression levels of MGAT3 between SKOV3 and SKOV3-ip cell lines. The results demonstrated that MGAT3 was differentially expressed between the two cell lines. The mRNA level of MGAT3 was significantly higher in SKOV3 cells than that in SKOV3-ip cells (61-fold, *p*<0.001, Figure 3A). Then the expression of MGAT3 in this two cell lines was further assessed at protein level by western blot analysis. The lower abundance of MGAT3 was observed for SKOV3-ip cells in contrast with SKOV3 cells (Figure 3B). Therefore, decreased MGAT3 expression in both mRNA and protein levels in SKOV3-ip cells were in accordance with decreased bisecting GluNAc modification in SKOV3-ip cell supernatant manifested by quantitative glycomics analysis.

### Effects of MGAT3 on Migration Ability of Ovarian Cancer Cells

**Figure 4:**
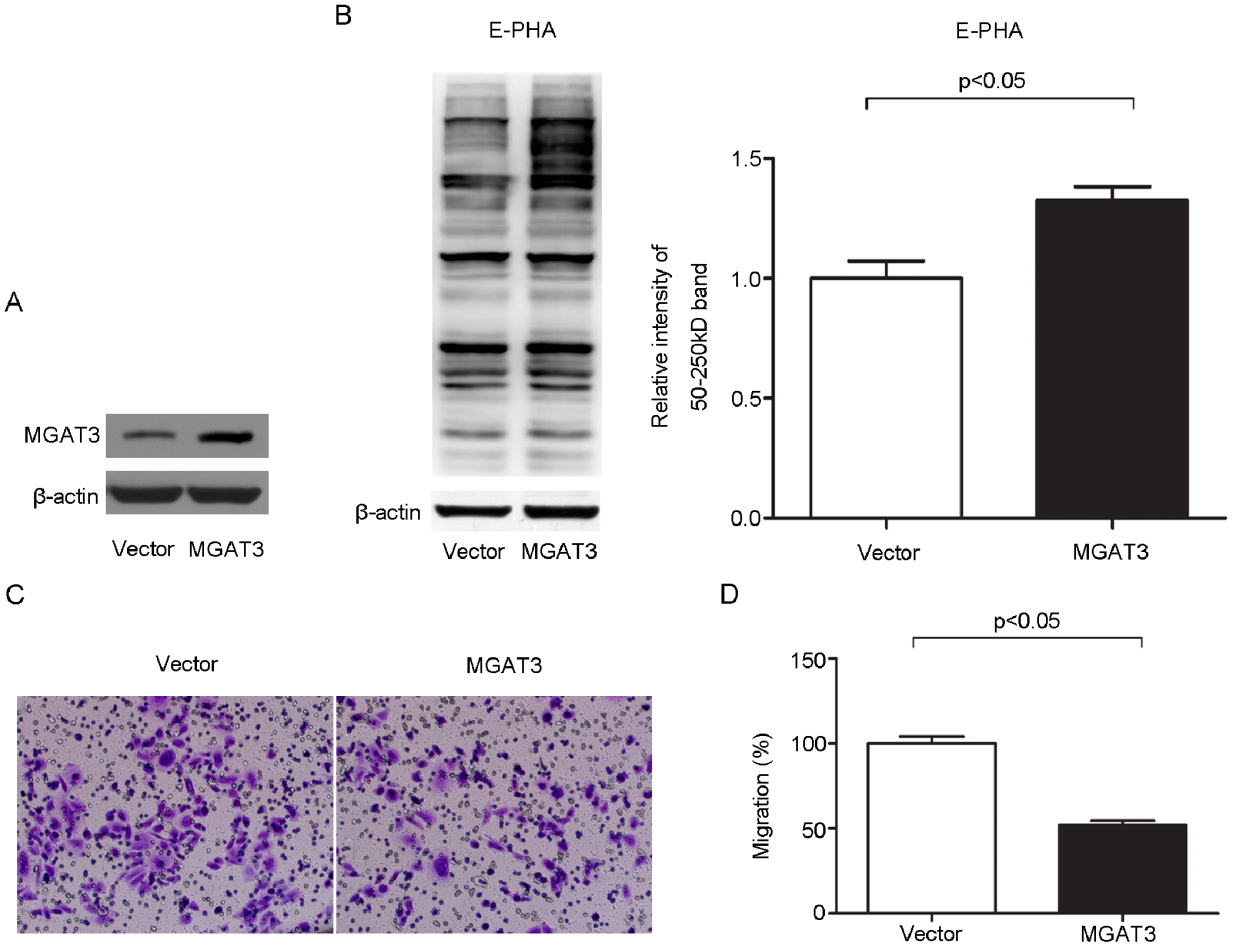
Overexpression of MGAT3 inhibits the migration ability of epithelial ovarian cancer cells. SKOV3-ip cells were transfected with pcDNA3.1 empty or MGAT3/pcDNA3.1 vectors. At 48 h after transfection, amounts of MGAT3 protein and its catalysate, *N*-glycans containing bisecting GlcNAc were detected by Western blotting (A) and Lectin blotting (B) in SKOV3-ip cells, respectively. Human beta-actin served as an endogenous control. All the relative expression levels of *N*-glycans containing bisecting GlcNAc were normalized to beta-actin. The left column of (B) represents the densitometry result for glycoproteins 50–250 kDa relative to control set at 1.0. It should be noted that the expression levels of MAGT3 and its catalysate, bisecting glycans were both significantly increased. Simultaneously, cells were subjected to the migration assay. Images of migrating cells from migration assay (C) and quantitative results are shown (D). Original magnification: 200x.The reported values are expressed as the means ± SEM. Each assay was performed at least three times. A *p*-value of less than 0.05 indicates statistical significance using Student’s *t*-test.

**Figure 5:**
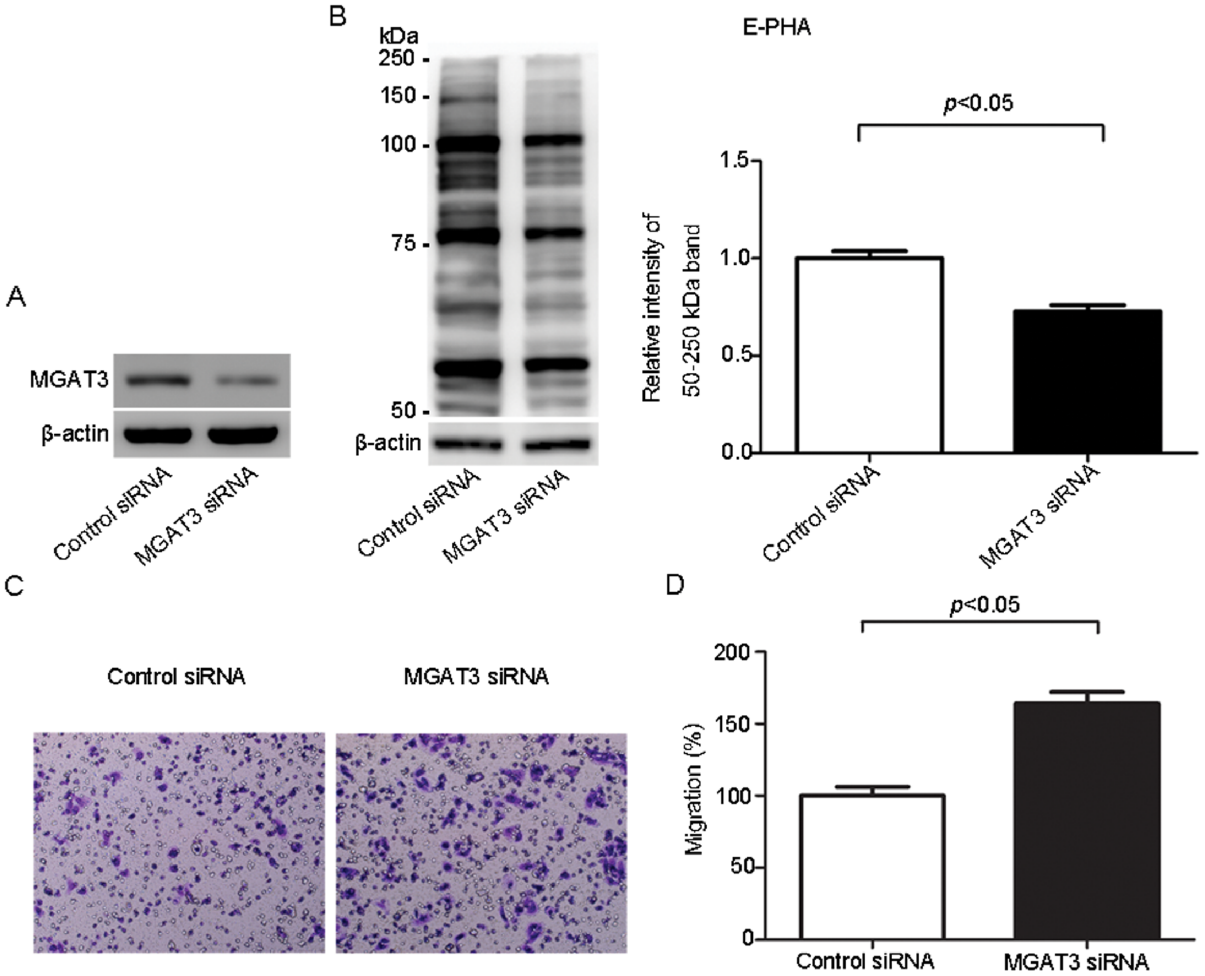
Knockdown of MGAT3 enhances the migration ability of epithelial ovarian cancer cells. SKOV3 cells were transfected with control siRNA and MGAT3-specific siRNA, respectively. At 48 h after transfection, amounts of MGAT3 protein and its catalysate, *N*-glycans containing bisecting GlcNAc were detected by Western blotting (A) and Lectin blotting (B) in SKOV3 cells, respectively. Human beta-actin served as an endogenous control. All the relative expression levels of *N*-glycans containing bisecting GlcNAc were normalized to beta-actin. The left column of (B) represents the densitometry result for glycoproteins 50–250 kDa relative to control set at 1.0. It should be noted that the expression levels of MAGT3 and its catalysate, bisecting glycans were both significantly decreased. Simultaneously, cells were subjected to the migration assay. Images of migrating cells from migration assay (C) and quantitative results are shown (D). Original magnification: 200x.The reported values are expressed as the means ± SEM. Each assay was performed at least three times. A *p*-value of less than 0.05 indicates statistical significance using Student’s *t*-test.

The data above showed that the levels of MGAT3 and its catalysate, *N*-glycans containing bisecting GlcNAc, were significantly lower in SKOV3-ip cells compared with that in SKOV3 cells. Since SKOV3-ip cells are the highly metastatic derivatives of SKOV3 cells, these results suggested a potential role of MGAT3 and its catalysate, bisecting glycans, on ovarian cancer cell motility. In order to test this, MGAT3 gene was overexpressed in SKOV3-ip cells by transfection. Western blot analysis demonstrated a significant increase in MGAT3 expression after transfection, compared with SKOV3-ip-control cells (Figure 4A). The level of bisecting glycans were also elevated accordingly (Figure 4B). To confirm whether overexpression of MGAT3 affect the migration ability, trans-well migration assays were performed. As shown in Figure 4C and 4D, migration ability in MGAT3 transfected cells was significantly decreased to 52% of that in control cells. Meanwhile, knockdown of MGAT3 in SKOV3 cells was performed by MGAT3-targeting siRNA transfection. Western blot analysis demonstrated efficient depression of MGAT3 expression in SKOV3 after transfection (Figure 5A). The level of bisecting glycans were down-regulated accordingly (Figure 5B). Trans-well migration assays (Figure 5C and 5D) showed 64% enhancement in migration ability in MGAT3-targeting siRNA transfected SKOV3 cells as compared with control cells. These results suggested that MGAT3 was involved in the migration ability of ovarian cancer cells, and may play a role in suppressing ovarian cancer cell metastasis.

### Immunohistochemical Analysis of MGAT3 Expression in Ovarian Cancer Tissues

**Figure 6:**
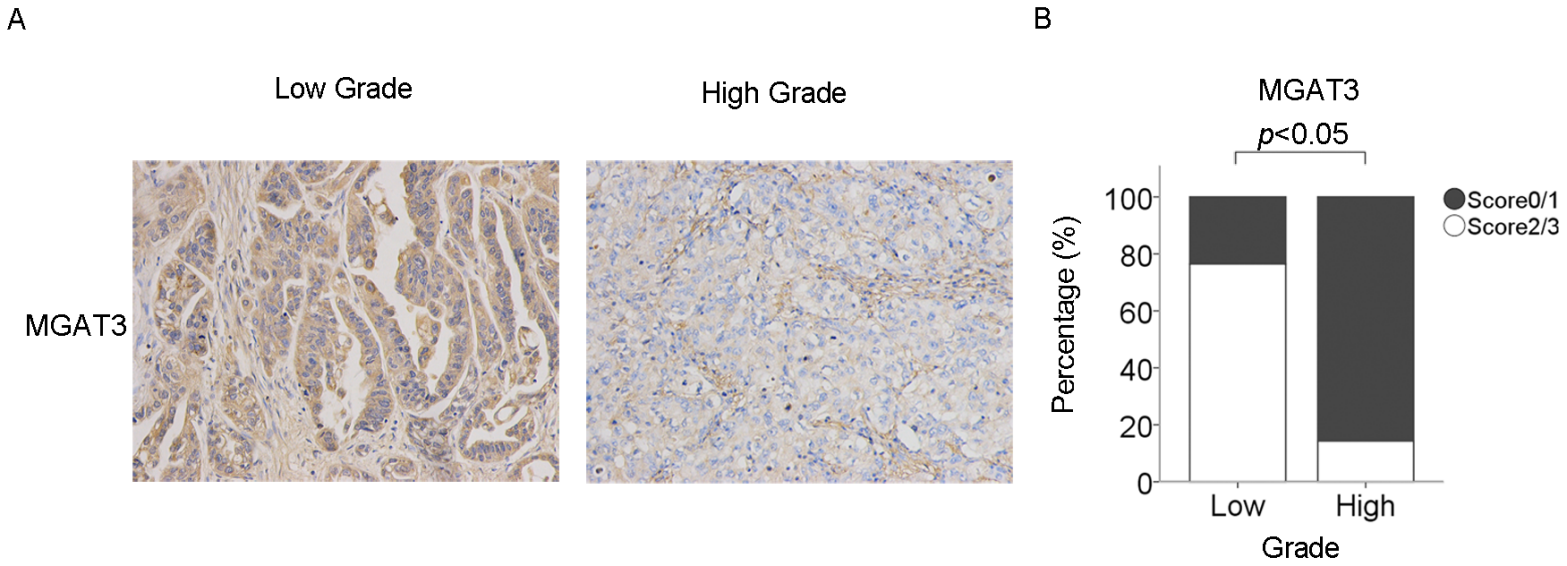
Expression of MGAT3 in human ovarian cancer tissues of varying grade (n = 24). (A) Representative immunohistochemistry photographs of MGAT3 expression in low grade and high grade ovarian cancer tissues. Original magnification: 200x. The immunostaining levels were scored as 0 (negative), 1 (weakly positive), 2 (moderately positive), or 3 (strongly positive). (B) Immunohistochemical staining was quantified using the scoring scale described above, as blindly determined by two pathologists. P value was calculated by Wilcoxon Signed Ranks Test and *p*<0.05 indicates statistical significance.

We further assessed MGAT3 expression in 24 ovarian cancer tissues employing immunohistochemical analysis. As shown in Figure 6, the expression levels of MGAT3 displayed a decreasing pattern, going from low grade ovarian cancer to high grade ovarian cancer. While only 14.29% of high grade ovarian cancer, 76.47% of low grade ovarian cancer specimens displayed moderate to strong staining of MGAT3. Immunohistochemical analysis indicated that lower MGAT3 expression correlated to ovarian cancers with higher malignant potential, which was in accordance with the results obtained from the SKOV3 cell line and its high metastatic derivative, SKOV3-ip cell lines.

## Discussion

In the present study, a comprehensive strategy was developed to facilitate the identification and validation of specific *N*-glycan changes associated with ovarian cancer metastasis. Accordingly, SKOV3 and its high metastatic derivative SKOV3-ip cells, which were isolated from ascites in the mouse given i.p. injections of SKOV3 cells [53], were chosen as experiment model. Notably, we aimed not only to discover the corresponding *N*-glycan alterations, but to confirm the featured change through multiple molecular biology approaches, including real-time PCR, western blot, lectin blot, transwell assay and immunohistochemistry analysis, as well.

From a clinical perspective, focusing on the glycosylation alteration of glycoproteins secreted by cancer cells is very appealing for diagnostic purposes, as they may filtrate into the peripheral blood [54], [55]. Discovery of biomarkers by direct analysis of blood plasma has been very difficult so far [55], [56], because plasma is a very complex fluid in which concentration of proteins are in high dynamic range. Therefore, the analysis of proteins secreted by homogeneous cancer cell populations has been reported as a more straightforward approach [57], [58], [59], [60]. Consequently, the conditioned medium of cell lines has widely been used for the analysis of cancer secretome [55], [61]. In this study, the differential *N*-glycosylation of secretome between SKOV3 and SKOV3-ip cells was investigated through global quantitative glycomic profiling of supernatant of these two cell lines based on metabolic incorporation of stable isotope labels [41]. In order to assess the changes of *N*-glycans in a reliable and sensitive manner, metabolic stable isotope labels were employed for MS-based quantitative glycomics analysis, which was reported capable of monitoring the glycan alterations associated with different biological conditions and diseases [40], [45], [62]. According to a recent research, a cell culture metabolic labeling strategy, IDAWG, was introduced, applying the principle of SILAC specialized for proteomic quantification to the glycomic quantification [41]. To be more specific, culture media containing amide-^15^N-Gln was used to metabolically label cellular aminosugars with heavy nitrogen, which was proved to be a simple implementation of quantitative tool for glycomic analysis [41]. Notably, in our study, this strategy was first utilized for the quantitative glycomics analysis of cell-secreted proteomes of ovarian cancer cells. Base on this method, changes in the structures and relative abundance of *N*-glycans of secreted glycoproteins from two ovarian cancer cells with different metastasis potential were obtained in a relatively reliable way. The results demonstrated that all the *N*-glycans with bisecting GlcNAc apparently decreased, while GlcNAc-branched *N*-glycans, high mannose structures increased in SKOV3-ip cell sceretome compared with that in SKOV3 cell secretome.

Bisecting GlcNAc residue is synthesized by a specific glycotransferase, *N*-acetylglucosaminyltransferase III (MGAT3) [63], which transfers GlcNAc in β1,4-linkage to the core Man of complex or hybrid *N*-glycans. MGAT3 is considered a key glycosyltransferase in *N*-glycan biosynthetic pathways and was reported playing an important role in tumor progression [52]. Knockdown of MGAT3 expression promoted MHCC97-L cells (human hepatocarcinoma cell line) invasion and increased resistance to 5-fluorouracil *in vitro* [64]. Transfection of MGAT3 in B16 melanoma cells resulted in suppressed tumor invasion and lung metastasis [65]. Song *et al.* reported that the bisecting GlcNAc on *N*-glycans inhibited growth factor signaling and retarded mammary tumor progression [32]. However, MGAT3 was reported upregulated in high metastatic human hepatocarcinoma cell line HCCLM3 in comparison with cell line with low metastatic potential [66]. Overexpression of MGAT3 in HeLaS3 cells enhanced the epidermal growth factor (EGF) mediated cell signaling [67], and transfecting myelogenous leukemia cell line K562 with MGAT3 protected it from natural killer cytotoxicity and increased spleen colonization [68]. The combined data indicates that effects of MGAT3 on tumor progression may vary with cell type.

In the case of ovarian cancer, relationship between bisecting glycan modification as well as MGAT3 and metastatic potential remains unclear. Abbott, K. L. *et al* reported increased expression of E-PHA binding proteins in endometrioid ovarian cancer tissue compared to normal ovarian tissue [69]. However, endometrioid ovarian cancer has been demonstrated to transform from endometriosis or ovarian epithelial cells [70]. Normal ovarian tissue, which is largely composed of ovarian stoma cells, could not be used as normal counterpart of ovarian cancer tissues. Our data from quantitative glycomics analysis suggested significantly decreased bisecting glycan modification in highly metastatic ovarian cancer cell line (SKOV3-ip). This finding was further supported by decreased expression of MGAT3 on both mRNA and protein levels observed in SKOV3-ip cells. Trans-well migration assays indicated that MGAT3 behaves as a suppressor in ovarian cancer cell migration. Immunhistochemistry analysis of MGAT3 expression in ovarian cancer tissues also showed correlation of over-expression of MGAT3 with lower metastatic potential, which was in line with the result from ovarian cancer cell lines.

One mechanism reported to be responsible for inhibitory effects of MGAT3 on cancer metastasis is that addition of bisecting GlcNAc by MGAT3 hinders β1,6 GlcNAc branching formation catalyzed by MGAT5, since MGAT5 cannot utilize the bisected oligosaccharide as an acceptor substrate [71], [72]. GlcNAc-branched *N*-glycans was reported to promote cancer cell metastasis via frequent sialic acid modifications on its remote saccharides and interaction with galectins to form lattices that regulate signaling receptor endocytosis [52]. As a consequence, increased expression of MGAT3 deceased β1,6 GlcNAc branching and metastatic potential of cancer cells. In this study, the antagonistic result of bisecting GlcNAc structures and GlcNAc-branched *N*-glycans was observed (Figures S3,S4). Increasing in β1,6 GlcNAc branching and MGAT5 expression in the highly metastatic SKOV3-ip cells were also manifested (Table 1, Figure S5 ), which were in accordance with findings reported in other cancers. Overexpression of MGAT5 in SKOV3 cells led to increased β1,6 GlcNAc branching and cell migration (Figure S6). These data suggested competitive effects of MGAT3 and MGAT5 on *N*-glycan processing in ovarian cancer. Notably, decreased MGAT3 expression and increased MGAT5 expression may be concurrent phenomenon that both contribute to increasedβ1,6 GlcNAc branching in highly metastatic ovarian cancer.

An important approach by which bisecting GlcNAc affects tumor cell behaviors is its modification of membrane or secreted glycoproteins that mediate cell-cell and cell-matrix interaction. E-cadherin is the main epithelial cell-cell adhesion molecule. In melanoma, MGAT3 upregulation, which in turn increased bisecting GlcNAc modification of E-cadherin [65], led to an enhancement of E-cadherin-mediated cell-cell interactions owing to prolonged turnover of E-cadherin on the cell surface [29], [73]. Introduction of bisecting *N*-glycan into integrin alpha(5)beta(1) inhibited cell migration on fibronectin [31]. Epidermal growth factor receptor (EGFR), a key signaling receptor for cell proliferation and migration, showed reduced binding with EGF and autophosphorylation after bisecting *N*-glycan modification in human U373 MG glioma cells [74] and neuronal cells [75]. Moreover, it has been reported that EGFR endocytosis enhanced in LEC10B CHO cells carrying bisected *N*-glycans, which led to downregulated EGFR signaling [32], [52]. Song *et al.* demonstrated that tumors and tumor-derived cells lacking Mgat3 exhibit enhanced signaling through the Ras pathway and reduced amounts of functionally glycosylated α-dystroglycan. Constitutive overexpression of an MMTV/Mgat3 transgene inhibits early mammary tumor development and tumor cell migration [32]. Interruption of lattice formation via branched *N*-glycans and galectin interaction was responsible for increased EGFR endocytosis [76], [77]. In the current study, inhibition of cell migration after MGAT3 transfection was also observed. E-cadherin, EGFR, and integrins, which are all important molecules for cell malignant transformation in ovarian cancer [78], [79], may also be modified by bisecting *N*-glycans. Change in their biological function or localization after bisecting *N*-glycan modification may contribute to ovarian cancer metastasis. Besides, molecules such as CA125 and CD44, which have been manifested to take part in adhesion of ovarian cancer cells to peritoneal membrane in a *N*-glycan dependent manner [35], [80], may also be the target of bisecting *N*-glycan modification. Further investigations employing lectin affinity chromatography and mass spectrometry are needed to fully uncover bisecting GlcNAc modified glycoproteins that are the effecting molecules in ovarian cancer metastasis.

## Conclusion

In this study, quantitative glycomics based on metablic stable isotopic labeling revealed a comprehensive picture of the metastasis-related *N*-glycans alteration in cell secreted proteome of epithelial ovarian cancer for the first time. Specifically, decrease in bisecting GlcNAc structure was found to be related to higher metastatic potential. Future studies will focus on discovery of specific ovarian cancer derived glycoproteins carring these metastasis-associated bisecting *N*-glycans to promote biomarker development for early detection of ovarian cancer.

## Supporting Information

Figure S1 **Representative tandem MS spectra of** ***N*** **-glycans containing bisecting GlcNAc.** (A) Tandem MS spectra of *m/z* 2012.7 [M+Na]^+^. (B) Tandem MS spectra of *m/z* 2377.8 [M+Na]^+^. N-glycans containning a bisecting GlcNAc residue were identified based on the Green circle indicates mannose; yellow circle indicates galactose; blue square indicates *N*-acetylglucosamine; red triangle indicates fucose. Asterisks (*) indicate signals of fragment ions losing water molecules.
(PDF)

Figure S2 **Representative MALDI mass spectrum profiles of quantitative analysis of** ***N*** **-glycans mixture.** (A) Unpermethylated *N*-glycans from a 1∶1 mixture of SKOV3-ip (amide-^14^N-Gln-labeled) and SKOV3 (amide-^15^N-Gln-labeled) supernatant. (B) Permethylated *N*-glycans from a 1∶1 mixture of SKOV3-ip (amide-^14^N-Gln-labeled) and SKOV3 (amide-^15^N-Gln-labeled) supernatant. The trend of alteration in all bisecting *N*-glycans are same whatever permethylation or not. While signal intensities were weakened and the profiles of *N*-glycans were more complex after permethylation. For instance, signal intensity of the unpermethylated *N*-glycan at an m/z value of 2012.7 was stronger than that of permethylated *N*-glycans at *m/z* values 2489.4, 2850.4, 3211.6 containing 0,1,2 sialic acids respectively. Green circle indicates mannose; yellow circle indicates galactose; blue square indicates *N*-acetylglucosamine; red triangle indicates fucose.
(PDF)

Figure S3 **Expression level of β1,6 GlcNAc branching** ***N*** **-glycans after MGAT3 overexpression in SKOV3-ip cell line.** SKOV3-ip cells were transfected with pcDNA3.1 empty or MGAT3/pcDNA3.1 vectors. At 48 h after transfection, amounts of MGAT3 protein and β1,6 GlcNAc branching *N*-glycans were detected by Western blotting (A) and Lectin blotting (B) in SKOV3-ip cells, respectively. Human beta-actin served as an endogenous control. All the relative expression levels ofβ1,6 GlcNAc branching *N*-glycans were normalized to beta-actin. The left column of (B) represents the densitometry result for glycoproteins 50–250 kDa relative to control set at 1.0. Each assay was performed at least three times. A *p*-value of less than 0.05 indicates statistical significance using Student’s t-test.
(PDF)

Figure S4 **Expression level of β1,6 GlcNAc branching** ***N*** **-glycans after MGAT3 knockdown in SKOV3 cell line.** SKOV3 cells were transfected with control siRNA and MGAT3-specific siRNA, respectively. At 48 h after transfection, amounts of MGAT3 protein andβ1,6 GlcNAc branching *N*-glycans were detected by Western blotting (A) and Lectin blotting (B) in SKOV3 cells, respectively. Human beta-actin served as an endogenous control. All the relative expression levels of β1,6 GlcNAc branching *N*-glycans were normalized to beta-actin. The left column of (B) represents the densitometry result for glycoproteins 50–250 kDa relative to control set at 1.0. Each assay was performed at least three times. A *p*-value of less than 0.05 indicates statistical significance using Student’s *t*-test.
(PDF)

Figure S5 **Differential expression of MGAT5 in SKOV3-ip and SKOV3 cell lines.** (A) The mRNA expression levels of MGAT5 in SKOV3-ip and SKOV3 cell lines. The mRNA levels of MGAT3 were normalized with GAPDH levels. Mean fold changes were calculated with the 2^-ΔΔCt^ method. (B) The protein expression levels of MGAT5 in SKOV3-ip and SKOV3 cell lines. Cell lysates were isolated by 10% SDS-PAGE for western blotting using antibody against MGAT5. Human beta-actin served as an endogenous control. Data are expressed as the means ± SEM. Each assay was performed at least three times. A *p*-value of less than 0.05 indicates statistical significance using Student’s *t*-test.
(PDF)

Figure S6 **Overexpression of MGAT5 enhances the migration ability of epithelial ovarian cancer cells.** SKOV3 cells were transfected with pcDNA3.1 empty or MGAT5/pcDNA3.1 vectors. At 48 h after transfection, amounts of MGAT5 protein and its catalysate, β1,6 GlcNAc branching *N*-glycans were detected by Western blotting (A) and Lectin blotting (B) in SKOV3 cells, respectively. Human beta-actin served as an endogenous control. All the relative expression levels ofβ1,6 GlcNAc branching *N*-glycans were normalized to beta-actin. The left column of (B) represents the densitometry result for glycoproteins 50–250 kDa relative to control set at 1.0. It should be noted that the expression levels of MAGT5 and its catalysate, β1,6 GlcNAc branching *N*-glycans were both significantly increased. Simultaneously, cells were subjected to the migration assay. Images of migrating cells from migration assay (C) and quantitative results are shown (D). Original magnification: 200x. The reported values are expressed as the means ± SEM. Each assay was performed at least three times. A *p*-value of less than 0.05 indicates statistical significance using Student’s *t*-test.
(PDF)

Table S1 **Clinical patient information for immunohistochemistry analysis.**
(DOC)
