# Supplementary figures and images for: Discovery of Specific Metastasis-Related N-Glycan Alterations in Epithelial Ovarian Cancer Based on Quantitative Glycomics
Source: PLoS One. 2014 Feb 6;9(2):e87978. doi: 10.1371/journal.pone.0087978 (PMC3916363; doi:10.1371/journal.pone.0087978)

**A**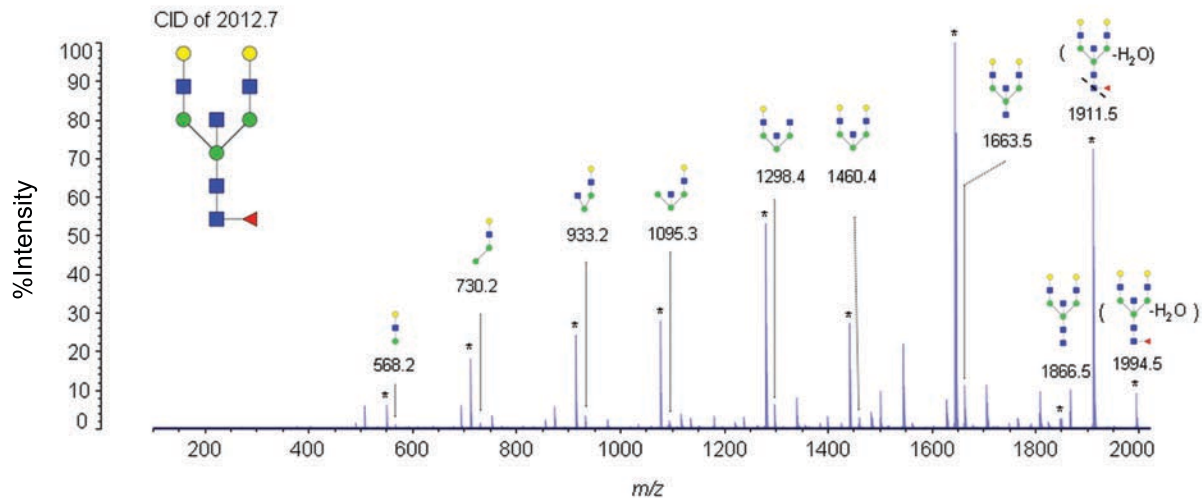**B**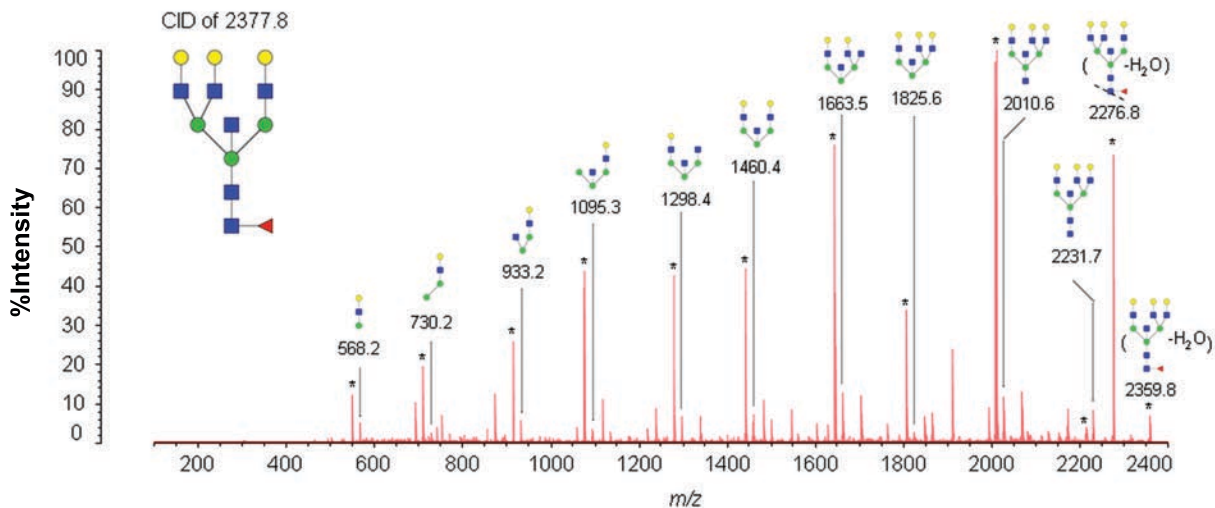

Supplement: Figure S1 — Representative tandem MS spectra of N -glycans containing bisecting GlcNAc. (A) Tandem MS spectra of m/z 2012.7 [M+Na]+. (B) Tandem MS spectra of m/z 2377.8 [M+Na]+. N-glycans containning a bisecting GlcNAc residue were identified based on the Green circle indicates mannose; yellow circle indicates galactose; blue square indicates N-acetylglucosamine; red triangle indicates fucose. Asterisks (*) indicate signals of fragment ions losing water molecules. (PDF) [file pone.0087978.s001.pdf]

A

Unpermethylation

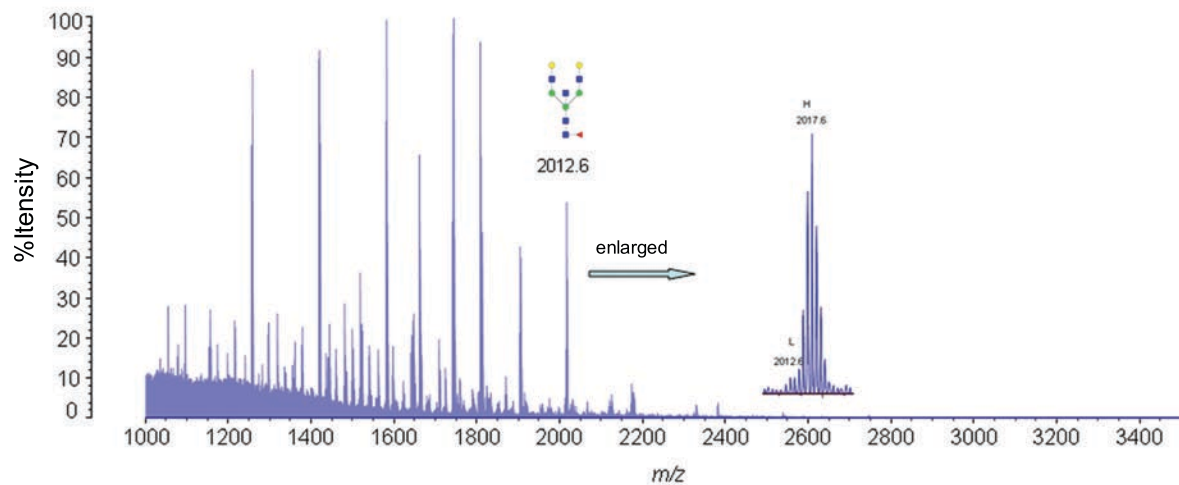

B

Permethylation

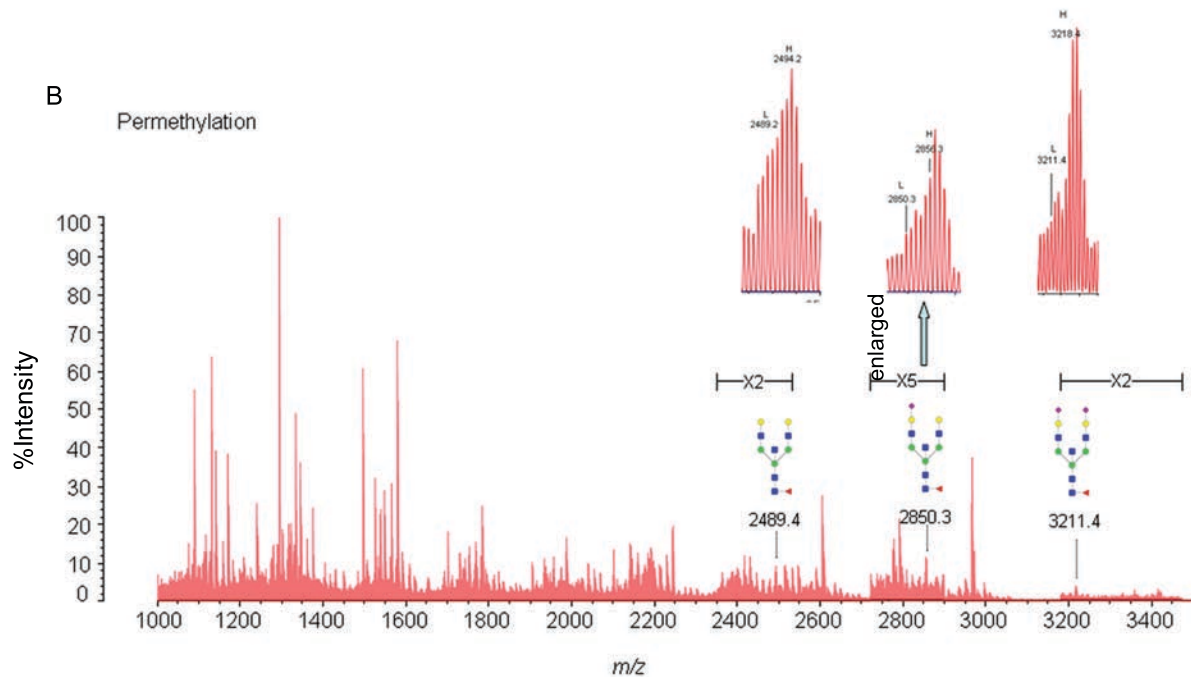

Supplement: Figure S2 — Representative MALDI mass spectrum profiles of quantitative analysis of N -glycans mixture. (A) Unpermethylated N-glycans from a 1∶1 mixture of SKOV3-ip (amide-14N-Gln-labeled) and SKOV3 (amide-15N-Gln-labeled) supernatant. (B) Permethylated N-glycans from a 1∶1 mixture of SKOV3-ip (amide-14N-Gln-labeled) and SKOV3 (amide-15N-Gln-labeled) supernatant. The trend of alteration in all bisecting N-glycans are same whatever permethylation or not. While signal intensities were weakened and the profiles of N-glycans were more complex after permethylation. For instance, signal intensity of the unpermethylated N-glycan at an m/z value of 2012.7 was stronger than that of permethylated N-glycans at m/z values 2489.4, 2850.4, 3211.6 containing 0,1,2 sialic acids respectively. Green circle indicates mannose; yellow circle indicates galactose; blue square indicates N-acetylglucosamine; red triangle indicates fucose. (PDF) [file pone.0087978.s002.pdf]

**A**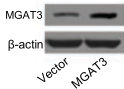**B**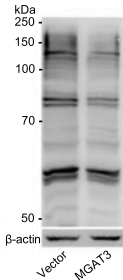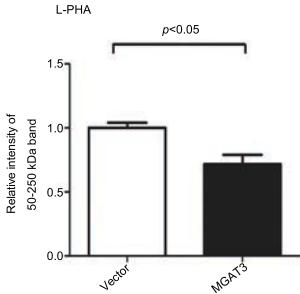

Supplement: Figure S3 — Expression level of β1,6 GlcNAc branching N -glycans after MGAT3 overexpression in SKOV3-ip cell line. SKOV3-ip cells were transfected with pcDNA3.1 empty or MGAT3/pcDNA3.1 vectors. At 48 h after transfection, amounts of MGAT3 protein and β1,6 GlcNAc branching N-glycans were detected by Western blotting (A) and Lectin blotting (B) in SKOV3-ip cells, respectively. Human beta-actin served as an endogenous control. All the relative expression levels ofβ1,6 GlcNAc branching N-glycans were normalized to beta-actin. The left column of (B) represents the densitometry result for glycoproteins 50–250 kDa relative to control set at 1.0. Each assay was performed at least three times. A p-value of less than 0.05 indicates statistical significance using Student’s t-test. (PDF) [file pone.0087978.s003.pdf]

A

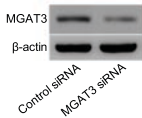

B

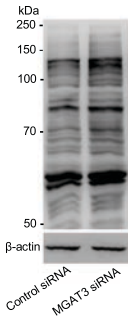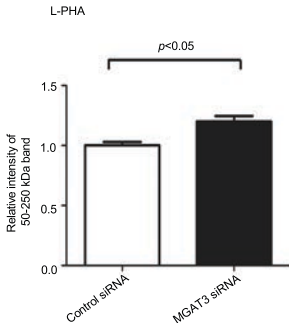

Supplement: Figure S4 — Expression level of β1,6 GlcNAc branching N -glycans after MGAT3 knockdown in SKOV3 cell line. SKOV3 cells were transfected with control siRNA and MGAT3-specific siRNA, respectively. At 48 h after transfection, amounts of MGAT3 protein andβ1,6 GlcNAc branching N-glycans were detected by Western blotting (A) and Lectin blotting (B) in SKOV3 cells, respectively. Human beta-actin served as an endogenous control. All the relative expression levels of β1,6 GlcNAc branching N-glycans were normalized to beta-actin. The left column of (B) represents the densitometry result for glycoproteins 50–250 kDa relative to control set at 1.0. Each assay was performed at least three times. A p-value of less than 0.05 indicates statistical significance using Student’s t-test. (PDF) [file pone.0087978.s004.pdf]

A

Relative mRNA levels

(MGAT5)

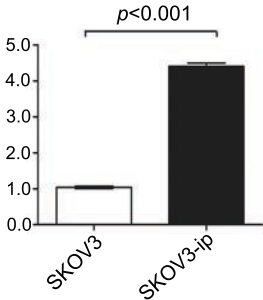

B

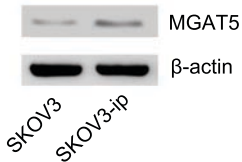

Supplement: Figure S5 — Differential expression of MGAT5 in SKOV3-ip and SKOV3 cell lines. (A) The mRNA expression levels of MGAT5 in SKOV3-ip and SKOV3 cell lines. The mRNA levels of MGAT3 were normalized with GAPDH levels. Mean fold changes were calculated with the 2-ΔΔCt method. (B) The protein expression levels of MGAT5 in SKOV3-ip and SKOV3 cell lines. Cell lysates were isolated by 10% SDS-PAGE for western blotting using antibody against MGAT5. Human beta-actin served as an endogenous control. Data are expressed as the means ± SEM. Each assay was performed at least three times. A p-value of less than 0.05 indicates statistical significance using Student’s t-test. (PDF) [file pone.0087978.s005.pdf]

A

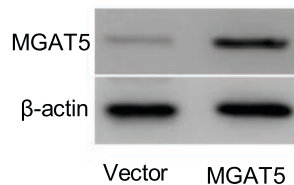

B

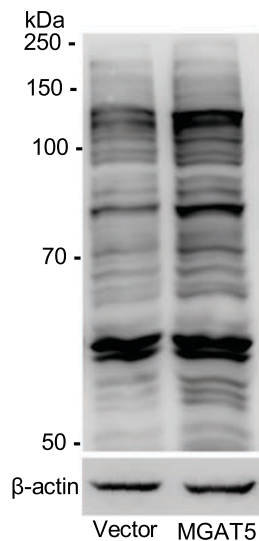

C

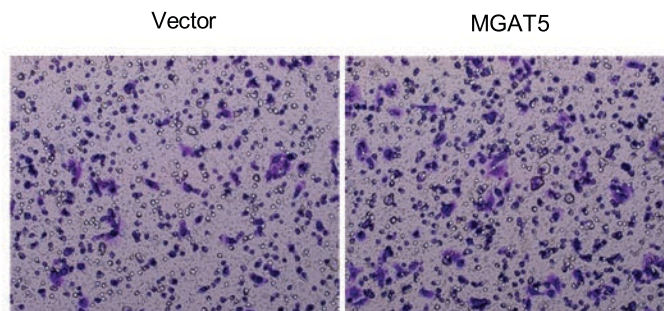

D

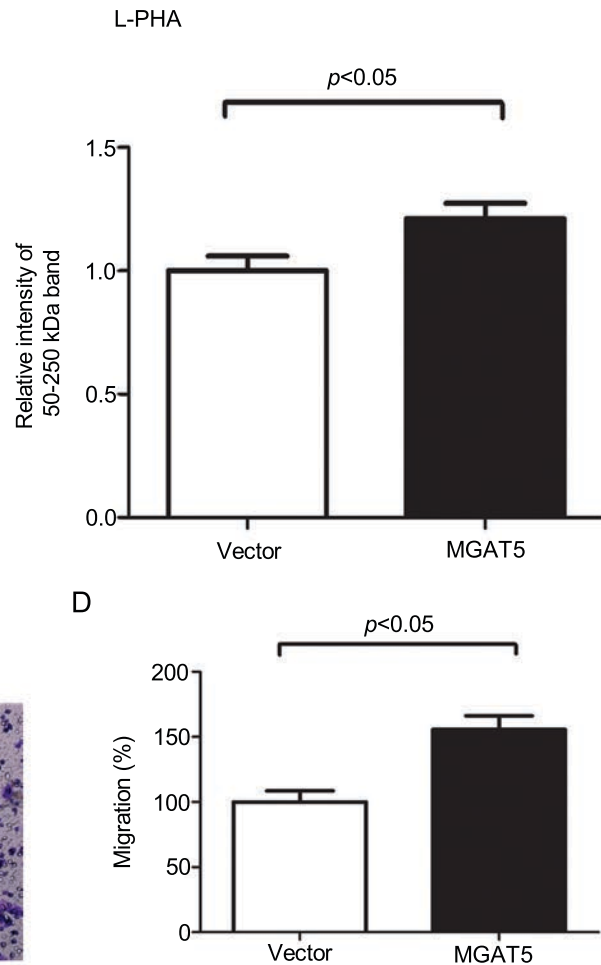

Supplement: Figure S6 — Overexpression of MGAT5 enhances the migration ability of epithelial ovarian cancer cells. SKOV3 cells were transfected with pcDNA3.1 empty or MGAT5/pcDNA3.1 vectors. At 48 h after transfection, amounts of MGAT5 protein and its catalysate, β1,6 GlcNAc branching N-glycans were detected by Western blotting (A) and Lectin blotting (B) in SKOV3 cells, respectively. Human beta-actin served as an endogenous control. All the relative expression levels ofβ1,6 GlcNAc branching N-glycans were normalized to beta-actin. The left column of (B) represents the densitometry result for glycoproteins 50–250 kDa relative to control set at 1.0. It should be noted that the expression levels of MAGT5 and its catalysate, β1,6 GlcNAc branching N-glycans were both significantly increased. Simultaneously, cells were subjected to the migration assay. Images of migrating cells from migration assay (C) and quantitative results are shown (D). Original magnification: 200x. The reported values are expressed as the means ± SEM. Each assay was performed at least three times. A p-value of less than 0.05 indicates statistical significance using Student’s t-test. (PDF) [file pone.0087978.s006.pdf]
