# Supplementary material for: Discovery of Specific Metastasis-Related N-Glycan Alterations in Epithelial Ovarian Cancer Based on Quantitative Glycomics
Source: PLoS One. 2014 Feb 6;9(2):e87978. doi: 10.1371/journal.pone.0087978 (PMC3916363; doi:10.1371/journal.pone.0087978)
Supplement: Table S1 — Clinical patient information for immunohistochemistry analysis. (DOC) [file pone.0087978.s007.doc]

| No. | Sex | Age | Organ | Pathology | Tumor Grade | Tumor Stage |
| --- | --- | --- | --- | --- | --- | --- |
| 1 | Female | 45 | ovary | serous adenocarcinoma | 1 | Ⅲc |
| 2 | Female | 49 | ovary | serous adenocarcinoma | 2 | Ⅲb |
| 3 | Female | 49 | ovary | serous adenocarcinoma | 1 | Ⅲc |
| 4 | Female | 23 | ovary | serous adenocarcinoma | 1 | Ic |
| 5 | Female | 23 | ovary | serous adenocarcinoma | 1 | Ic |
| 6 | Female | 42 | ovary | serous adenocarcinoma | 2 | Ⅲc |
| 7 | Female | 43 | ovary | serous adenocarcinoma | 1 | Ⅲc |
| 8 | Female | 61 | ovary | serous adenocarcinoma | 2 | Ia |
| 9 | Female | 23 | ovary | serous adenocarcinoma | 2 | Ic |
| 10 | Female | 56 | ovary | serous adenocarcinoma | 3 | Ⅲc |
| 11 | Female | 68 | ovary | serous adenocarcinoma | 3 | Ⅲb |
| 12 | Female | 42 | ovary | serous adenocarcinoma | 3 | Ⅲc |
| 13 | Female | 61 | ovary | serous adenocarcinoma | 3 | Ⅲc |
| 14 | Female | 69 | ovary | serous adenocarcinoma | 2 | Ⅲc |
| 15 | Female | 60 | ovary | serous adenocarcinoma | 1 | Ic |
| 16 | Female | 43 | ovary | serous adenocarcinoma | 2 | Ⅲc |
| 17 | Female | 57 | ovary | serous adenocarcinoma | 2 | Ic |
| 18 | Female | 56 | ovary | serous adenocarcinoma | 3 | Ⅲc |
| 19 | Female | 45 | ovary | serous adenocarcinoma | 3 | Ⅲc |
| 20 | Female | 59 | ovary | serous adenocarcinoma | 2 | Ⅲb |
| 21 | Female | 54 | ovary | serous adenocarcinoma | 2 | Ⅲb |
| 22 | Female | 51 | ovary | serous adenocarcinoma | 2 | Ⅲc |
| 23 | Female | 58 | ovary | serous adenocarcinoma | 1 | Ia |
| 24 | Female | 28 | ovary | serous adenocarcinoma | 3 | Ic |
